# *Dorstenia triseriata* (Moraceae) a new and endangered species from Brazil

**DOI:** 10.3897/phytokeys.38.7086

**Published:** 2014-05-19

**Authors:** Anderson F. P. Machado, Jorge Fontella Pereira, J. Pedro P. Carauta

**Affiliations:** 1 Programa de Pós-graduação em Botânica, Universidade Estadual de Feira de Santana, Avenida Transnordestina s/n°, Novo Horizonte, 44036-900 Feira de Santana, BA, Brazil; 2 Departamento de Botânica, Museu Nacional/Universidade Federal do Rio de Janeiro, Quinta da Boa Vista s/n°, São Cristóvão, 20940-040, Rio de Janeiro, RJ, Brazil

**Keywords:** Atlantic rainforest, Dorsteniae, neotropics, taxonomy

## Abstract

A new species of Moraceae is described, illustrated and compared to its close morphological relatives. *Dorstenia triseriata* presents similarities with *Dorstenia turnerifolia* but distinguished by size of peduncle, diameter of receptacle, number of bract rows, color of marginal bracts, and by an indistinct fringe on inflorescence. A conservation assessment based on IUCN criteria determines the new species to be vulnerable (VU).

## Introduction

*Dorstenia* L. currently includes approximately 105 species and is the second largest genus of Moraceae (Berg 2001). This genus has a mostly herbaceous habit, marked by the absence of tepals in the pistillate flowers, simple interfloral bracts, and an expanded receptacle (the coenanthium) containing many grouped, diminutive flowers (De Granville 1971, Berg 2001).

*Dorstenia* sect. Lecania Fisher & Meyer comprises approximately 24 species, which are endemic to the rainforests of eastern Brazil. The species of section Lecania are characterized by herbaceous to suffrutescent plants usually with unbranched stems, long internodes, broad to subulate stipules, and inflorescences mostly orbicular to elliptical in shape (Carauta 1978, Berg 2001). The new species described here belongs to this section according to description adopted by Carauta (1978) and Berg (2001).

## Taxonomic treatment

### Dorstenia triseriata

A.F.P.Machado, Fontella & Carauta sp. nov.

urn:lsid:ipni.org:names:77138784-1

http://species-id.net/wiki/Dorstenia_triseriata

#### Type.

**Figure 1.**
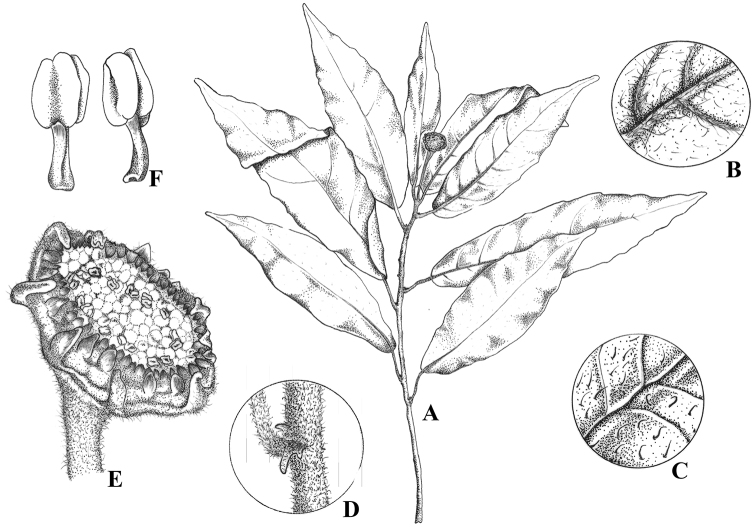
*Dorstenia triseriata*. **A** Habit **B** Leaf abaxial detail **C** Leaf adaxial detail **D** stipule **E** Inflorescence receptacule **F** Stamens (based on Carrijo & Machado 1508, Holotype).

**Figure 2.**
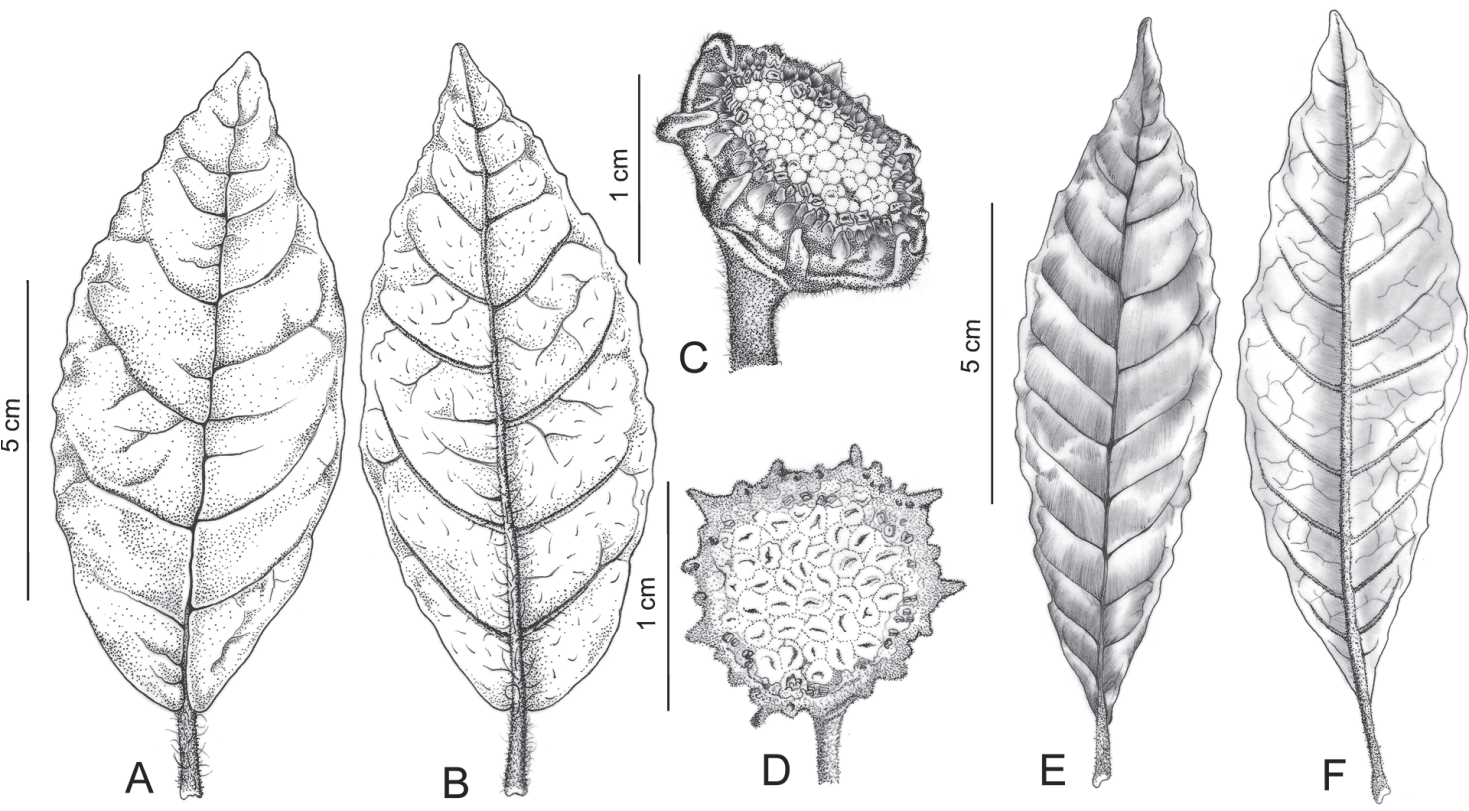
*Dorstenia triseriata*. **A** leaf adaxial surface **B** leaf abaxial surface **C** Inflorescence lateral view. *Dorstenia turnerifolia* **D** Inflorescence **E** leaf adaxial surface **F** leaf abaxial surface. (**A–C** based on Carrijo & Machado 1508; **D–F** based on A.F.P. Machado 1081, HUEFS).

Brazil. Espírito Santo: Município de Santa Teresa, Parque Natural Municipal São Lourenço, 19°56'09"S, 40°36'00"W. 28 oct 2008. TT Carrijo 1508 & AFP Machado (holotype: R!) Fig. 1A–F; 2A–C.

Dorsteniae turnerifoliae similis, sed pedunculo elongato (in vicem brevi), receptaculo rotundato cum una serie bracteis submarginalibus viridibus etiam duabus seriebus bracteis marginalibus violaceis (nec tantum una serie bracteis) et fimbria non distincta (in vicem fimbria distincta) differt.

Herb to subshrub up to 40 cm tall; rhizome short; stem 3–5 mm thick, hirtellous with straight and uncinate hairs, internodes up to 1(-2.0) cm long. Leaves spiraled, lamina oblong to lanceolate, or subovate to subobovate, 3–7(-9) × 1.5(-2.5) cm long, chartaceous, apex acute, base rounded, margin dentate; indumentum rough on both surfaces, of uncinate hairs, plus simple and elongated hairs on veins, lateral veins 5–8 pairs, prominent on the abaxial surface, tertiary venation reticulate; petiole 1–2 cm long, pilose; stipules persistent, subulate, 1–2 mm long, coriaceous, deflexed. Inflorescences greenish, margin purplish; peduncle 1.0–3.5 cm long, pubescent with simple and elongated hairs; receptacle circular, 0.3–0.8 cm in diameter, margin dentate, fringe not distinct; submarginal bracts green in 1 row, ovate, up to 0.5 mm long; marginal bracts purplish in 2 rows; staminate flowers at the periphery of inflorescence, tepals 3, stamens 2, filaments as long as the perianth; pistillate flowers sessile, tepals 2–3, ovary free, stigmas 2 subequal in length; fruit a dehiscent drupe rough, 2 mm.

#### Etymology.

The specific epithet is an allusion to number of marginal bracts rows, arranged in three vertical rows.

#### Distribution and habitat.

*Dorstenia triseriata* inhabits the states of Espírito Santo, Bahia, and Minas Gerais in Brazil.

#### Observations.

*Dorstenia triseriata* is closely related to two other species: *Dorstenia romaniucii* A.F.P.Machado & M.D.M. Vianna and *Dorstenia turnerifolia* Fisch. & C.A. Mey. From *Dorstenia romaniucii* both other species are differentiated by the staminate flowers disposed peripherally at the inflorescence. *Dorstenia triseriata* is distinguished from *Dorstenia turnerifolia* by its elongated peduncle (vs. short), purplish and circular receptacles (vs. green and angular receptacles), fringe not distinct (vs. fringe distinct) and marginal bracts in three rows 1 green and 2 purplish (vs. 1 row). submarginal bracts green in 1 row, ovate, up to 0.5 mm long; marginal bracts purplish in 2 rows

The circumscription of *Dorstenia turnerifolia* adopted by Berg and Hijman (1999) and Berg (2001) is too broad and covers many taxa. However, the analysis of classical collections, original description, and the plant material collected by the first author at the type locality allows the recircumscription of *Dorstenia turnerifolia* (Fig. 2D, E, F and Table 1) and allows us to recognize *Dorstenia triseriata* as a new species.

**Table 1.** Comparative morphology of *Dorstenia triseriata* and its related species.

Characters	Leaf blade (cm long)	Lateral veins	Peduncle (cm)	Receptacle	Marginal bracts (rows)	Fringe
*Taxa*						
*Dorstenia triseriata*	3–7(–9)	5–8 pairs	1.5–3.0	Rounded (0.3–0.8)	3 (1 green + 2 purple)	Not distinct
*Dorstenia romaniucii*	2–13(–17)	14–16 pairs	1–1.5	slightly angular (1–1.3)	2 (green)	Not distinct
*Dorstenia turnerifolia*	5–7(–17)	(7–)10–17 pairs	0.5–1.0	Orbicular to rounded (1–2)	1 (green)	Distinct

In *Dorstenia* the vegetative characters can be very variables specially when observed in a herbarium material. However, the reproductive characters are most reliable. The new species belongs to a group of species with similar habit and similar vegetative structures. The specimens of this group are commonly identified at herbaria as *Dorstenia turnerifolia*. Despite this they can easily separated with a criterious analysis of reproductive structures. The main differences between these taxa are also showed in Fig. 2 and Table 1.

#### Conservation status.

According to IUCN (2001) criteria, this species is considered Vulnerable (VU B2a; B2bi, ii, iii). Area of occupancy estimated to be less than 2000 Km² with a decrease in area of occupancy, extension of occurrence, and habitat quality.

#### Key of *Dorstenia triseriata* and its related species

**Table:** 

1	Receptacle slightly angular with two rows of marginal bracts. Staminate flowers intermixed with pistillate flowers	*Dorstenia romaniucii*
–	Receptacle rounded or orbicular. Marginal bracts in 1 or 3 rows. Staminate flowers grouped peripherally at the inflorescence	2
2	Peduncle 0.5–1 cm, receptacle 1–2 cm diam. Marginal bracts green disposed in one row. Fringe distinct	*Dorstenia turnerifolia*
–	Peduncle 1–3.5 cm, receptacle 0.3–0.8 cm diam. Marginal bracts disposed in three rows (1 green + 2 purplish). Fringe not distinct	*Dorstenia triseriata* sp. nov.

#### Additional specimens examined.

Brazil. Espírito Santo. Santa Teresa, 28 May 2008, *Machado* 937 (R); l.c., Santa Teresa, Parque Natural Municipal São Lourenço, 29 Mar. 2007, *Monteiro et al.* 368 (R); l.c., Santa Teresa, Parque Natural Municipal São Lourenço, *Machado* 936, l.c., Santa Teresa, *Carrijo*, 1507. May 2008 (R). Bahia. Itanhaém, 17°8'17"S, 40°25'34"W. 29 Dec 2004, *Amorim et al.*, 4629 (HUEFS). Minas Gerais. Itabira, Fazenda do Quilombo, 19°37'10"S, 43°13'36"W. 27 Jan. 1943, *Magalhães s.n.* (HUEFS 118735)

## Supplementary Material

XML Treatment for Dorstenia triseriata

## References

[B1] Berg CC Hijman MEE (1999) The genus *Dorstenia* (Moraceae). Ilicifolia 2: 1−211

[B2] Berg CC (2001) Moreae, Artocarpeae and *Dorstenia* (Moraceae): with introductions to the family and *Ficus* and with additions and corrections to Flora Neotropica 7, Mon. 83. New York Botanical Garden, New York, 1−346

[B3] Carauta JPP (1978) *Dorstenia* L. (Moraceae) do Brasil e países limítrofes. Rodriguésia 29(44): 53–223; fig. 1−41

[B4] De Granville JJ (1971) Notes sur la Biologie Florale de quelques Espèces du Genre *Dorstenia* (Moracées). Office of the Scientific and Technical Research Overseas, Série Biologie 15: 61−97

[B5] IUCN (2001) IUCN Red List Categories and Criteria, Version 3.1. www.iucn.org

